# 

**Published:** 1881-09

**Authors:** 


### CONTENTS.

### COMMUNICATIONS.

The Study of Dental Surgery and the Means Thereto ........ 355
Address by Dr. Edwards ........ 369
International Medical Congress ........ 374
Deciduous Teeth ........ 379
CORRESPONDENCE ........ 382

### SELECTIONS.

Cholera Infantum ........ 383
Surgery—Syphilitic Alterations of the Teeth ........ 386
Removal of a double-pointed Needle from the Submaxillary Connective Tissue, by the Aid of Manipulation ........ 388
What are High Prices ........ 389
Adhesive Wax ........ 390
Translations ........ 391
A Case of Abscess in the Superior Maxillary Sinus not due to a Diseased Tooth ........ 391
New Instruments for the Engine by means of which Gold Fillings can be Rapidly and Securely Inserted ........ 393
The Haering Patent ........ 393
Howard's Method of Artificial Respiration ........ 394
Taking a Bite ........ 394

### PROCEEDINGS.

The Goodyear Patent ........ 395
Tincture of Chloride of Iron ........ 395
Northern Ohio Dental Association ........ 395

### EDITORIAL.

American Dental Association ........ 397
A New Arm Rest ........ 398
Exemption from Jury Duty ........ 398

